# Comparative proteomics analysis of oral cancer cell lines: identification of cancer associated proteins

**DOI:** 10.1186/1477-5956-12-3

**Published:** 2014-01-15

**Authors:** Saiful Anuar Karsani, Nor Afiza Saihen, Rosnah Binti Zain, Sok-Ching Cheong, Mariati Abdul Rahman

**Affiliations:** 1 Institute of Biological Sciences, Faculty of Science, University of Malaya, 50603 Kuala Lumpur, Malaysia; 2 Oral Cancer Research and Co-ordinating Centre & Faculty of Dentistry, University of Malaya, 50603 Kuala Lumpur, Malaysia; 3 Oral Cancer Research Team, 2nd Floor Outpatient Centre, Sime Darby Medical Centre, Cancer Research Initiatives Foundation (CARIF), 47500 Subang Jaya, Selangor, Malaysia; 4 Department of Clinical Oral Biology, Faculty of Dentistry, Universiti Kebangsaan Malaysia, 50300 Kuala Lumpur, Malaysia; 5 Department of Oral and Maxillofacial Surgery, Faculty of Dentistry, University of Malaya, 50603 Kuala Lumpur, Malaysia; 6 University of Malaya Centre for Proteomics Research (UMCPR), University of Malaya, 50603 Kuala Lumpur, Malaysia

**Keywords:** Oral cancer, Proteomics, Cell lines, Cancer associated proteins

## Abstract

**Background:**

A limiting factor in performing proteomics analysis on cancerous cells is the difficulty in obtaining sufficient amounts of starting material. Cell lines can be used as a simplified model system for studying changes that accompany tumorigenesis. This study used two-dimensional gel electrophoresis (2DE) to compare the whole cell proteome of oral cancer cell lines vs normal cells in an attempt to identify cancer associated proteins.

**Results:**

Three primary cell cultures of normal cells with a limited lifespan without hTERT immortalization have been successfully established. 2DE was used to compare the whole cell proteome of these cells with that of three oral cancer cell lines. Twenty four protein spots were found to have changed in abundance. MALDI TOF/TOF was then used to determine the identity of these proteins. Identified proteins were classified into seven functional categories – structural proteins, enzymes, regulatory proteins, chaperones and others. IPA core analysis predicted that 18 proteins were related to cancer with involvements in hyperplasia, metastasis, invasion, growth and tumorigenesis. The mRNA expressions of two proteins – 14-3-3 protein sigma and Stress-induced-phosphoprotein 1 – were found to correlate with the corresponding proteins’ abundance.

**Conclusions:**

The outcome of this analysis demonstrated that a comparative study of whole cell proteome of cancer versus normal cell lines can be used to identify cancer associated proteins.

## Background

Oral cancer is a devastating disease that ranks as the fifth most common type of cancer affecting humans worldwide [1]. Incidence and mortality rates vary widely across the world. There are approximately 500,000 new oral and pharyngeal cancer cases diagnosed annually, with three quarters being registered in developing countries [2,3]. The disease is highly associated with established cultural risk factors such as tobacco chewing/smoking, alcohol consumption and betel-quid chewing [4]. In western countries, cigarette smoking and alcohol drinking are the major risk factors while betel-quid chewing and smoking are the major risk factors that contribute to the development of oral cancer in South Asia, Southeast Asia and Taiwan [1]. Oral cancer appears as an abnormal growth within the mouth region and this include the buccal mucosa (cheek), tongue, floor of the mouth and lip. Oral squamous cell carcinoma (OSCC) represents the highest of all oral malignancy, accounting for more than 95% of total cases reported [1,3].

There is a dearth of knowledge with regards to the development and progression of oral cancer. The exact molecular mechanisms remain unknown. However, it is known to involve the activation of oncogenes, change in expression of various proteins which would eventually lead to the development of cancer. Thus, a study of proteins that change in oral cancer will provide valuable information and add to our understanding of the disease. It may also identify candidate proteins that can potentially be utilized as biomarkers for early detection. The ability of proteomics to compare differences/changes in proteome profiles (including changes in post-translational modifications) which are related to tumor progression has been adapted in clinical research for the identification of biomarkers for disease such as cancer [5].

One of the main problems in performing proteomics analysis on cancerous cells is the difficulty in obtaining sufficient amounts of material to perform the analysis. The availability of cancer cell lines makes it possible to obtain an almost limitless amount of sample for proteomic analysis. Cultured cancer and normal cell lines can be used as a simplified model system for studying changes that accompany tumorigenesis. Thus, this study aims to utilize proteomics in identifying differences between proteome profiles of normal primary cultures vs oral cancer cell lines. Our results showed that such an analysis will identify cancer associated proteins.

## Results and discussions

### Identification of proteins with different abundance between normal primary cultures and cancer cell lines

**Figure 1:**
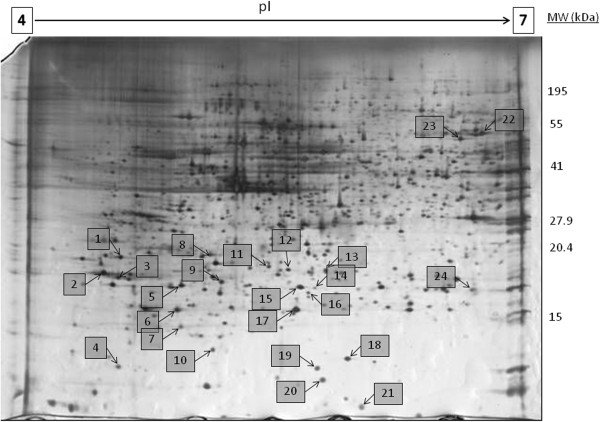
**Representative 2DE gel of whole cell proteome of cell lines.** The whole cell protein of normal primary cultures and cancer cell lines were resolved by 2DE pI 4–7, 11% second dimension polyacrylamide gel. Proteins with different abundance are shown as numbered spots. The numbers correspond to Spot Number in Table 1.

More than 1000 individual protein spots were resolved and visualized on silver stained gels. A representative gel is shown in Figure 1. Image analysis identified 24 protein spots that exhibited significant difference in abundance (*p* < 0.05). All 24 were unambiguously identified by MALDI-TOF/TOF MS and their respective identities are shown in Table 1. The index numbers in Table 1 correspond to the numbers in Figure 1 and they indicate the location of the proteins on the 2DE gels. From this point forward, proteins will be referred to by their abbreviated names as shown in Table 1. Table 1 includes zoomed images for all protein spots. The identified proteins were classified into seven functional categories – structural proteins, enzymes, regulatory proteins, chaperones and others. For almost all identified proteins, the experimental and theoretical pI and molecular weights were matched.

**Table 1:** List of proteins with different abundance in cancer cell lines

**Protein name**		**Ascension number (Swissprot)/matched 2D coordinates**	**MW/pI**		**Peptides (% cov)**	**Relative change vs normal**	**Zoomed in gel image showing representative spots**	
**Abbreviation**		**(World-2DPAGE portal)**						
			**Theory**	**Experimental**			**Normal**	**Cancer**
Structural								
20	Stathmin (Phosphoprotein p19)	P16949 /SPOT 2D-001YH8	17.3/5.76	>15.0/5.7	4 (18%)	+1.6		
	STMN1							
1	Tropomyosin alpha-3 chain (Tropomyosin-3)	P06753 /	32.8/4.68	>30.0/4.6	6 (13%)	-1.5		
		SPOT 2D-001EM1						
		SPOT 2D-001YBH						
	TPM3	SPOT 2D-001JNO						
4	Myosin regulatory light chain-2	P19105 /	19.8/4.67	>15.0/4.60	5 (17%)	-1.9		
		SPOT 2D-001JUH						
	ML12A	SPOT 2D-001JUS						
Enzymes								
6	Lactoylglutathione lyase (Glyoxalase I). (EC 4.4.1.5)	Q04760 /	20.8/5.12	>20.0/5.00	10 (22%)	+7.7		
	LGUL	n/a						
24	Triosephosphate isomerase (EC 5.3.1.1)	P60174 /	26.7/6.45	>25.0/6.5	5 (28%)	+2.5		
	TPIS	SPOT 2D-0003MP						
18	Nucleoside diphosphate kinase A (EC 2.7.4.6)	P15531 /	17.1/5.83	>15.0/5.8	8 (31%)	+1.5		
	NDKA	n/a						
21	Ubiquitin-conjugating enzyme E2 N (EC 6.3.2.19)	P61088 /	17.1/6.13	>15.0/5.9	6 (23%)	+2.6		
	UBE2N	n/a						
16	Gamma-glutamylcyclotransferase (EC 2.3.2.4)	O75223 /	21.0/5.07	>20.0/5.0	3 (27%)	+2.5		
	GGCT	n/a						
14	Peroxiredoxin-4 (EC 1.11.1.15)	Q13162 /	30.5/5.86	>30.0/5.8	6 (16%)	+2.2		
	PRDX4	n/a						
19	Superoxide dismutase [Cu-Zn] (EC 1.15.1.1)	P00441 /	15.9/5.70	>15.0/5.70	5 (24%)	+1.4		
		SPOT 2D-001F1V						
	SODC	SPOT 2D-000ZXR						
17	Glutathione S-transferase P (GST class-pi). (EC 2.5.1.18)	P09211 /	23.3/5.43	>25/5.6	8 (22%)	+1.4		
		SPOT 2D-001EUM						
		SPOT 2D-0003Q9						
	GSTP1	SPOT 2D-00085X						
11	Proteasome subunit beta type 4 precursor. (EC 3.4.25.1)	P28070 /	29.2/5.72	29.0/5.7	6 (15%)	+1.6		
		SPOT 2D-000851						
	PSB4	SPOT 2D-000D4M						
Regulatory proteins								
3	14-3-3 protein beta/alpha	P31946 /	28.0/4.70	>25.0/4.50	6 (4%)	+1.8		
	1433B	n/a						
12	Prohibitin	P35232 /	29.8/5.57	>25.0/5.60	5 (12%)	+1.6		
	PHB	SPOT 2D-001EOL						
13	Proteasome activator complex subunit 1	Q06323 /	28.7/5.78	>25.0/5.70	2 (4%)	-1.7		
	PSME1	SPOT 2D-001YC7						
16	Proteasome activator complex subunit 2	Q9UL46 /	27.3/5.44	>30.0/5.4	4 (11%)	-2.3		
	PSME2	n/a						
2	14-3-3 protein sigma (Stratifin)	P31947 /	27.8/4.68	>25.0/4.50	9 (24%)	-2.0		
	1433S	SPOT 2D-001EP6						
22	Stress-induced-phosphoprotein 1	P31948 /	62.6/6.4	>55.0/6.50	11 (12%)	+2.1		
	STIP1	n/a						
10	Interleukin-1 receptor antagonist protein precursor	P18510 /	20.0/5.83	>20.0/5.3	2 (8%)	+1.3		
	IL1RA	n/a						
5	Rho GDP-dissociation inhibitor 1	P52565 /	23.2/5.02	30.0/5.0	4 (14%)	+2.6		
	GDIR	n/a						
9	Ran-specific GTPase-activating protein	P43487 /	23.3/5.19	30/5.2	5 (18%)	+1.8		
	RANG	n/a						
Chaperones								
15	Heat-shock protein beta-1	P04792 /	22.8/6.00	>20.0/5.8	11 (32%)	+3.1		
		SPOT 2D-001JQI						
	HSPB1	SPOT 2D-001JQP						
23	T-complex protein 1 subunit zeta-2	Q92526 /	57.7/6.63	>55.0/6.8	2 (2%)	-1.6		
	TCPW	n/a						
Others								
8	Chloride intracellular channel protein 1 (membrane associated)	O00299 /	26.9/5.09	>30.0/5.1	14 (46%)	-1.7		
	CLIC1	SPOT 2D-001YBL						

The proteins were grouped into five functional categories – structural proteins, enzymes, regulatory proteins, chaperones and others. The accession number (swiss-prot) and matched 2D coordinates, molecular weight and pI (theoretical and experimental), number of matched peptides, percentage coverage, relative change vs normal and zoomed in gel image showing representative spots are shown.

**Figure 2:**
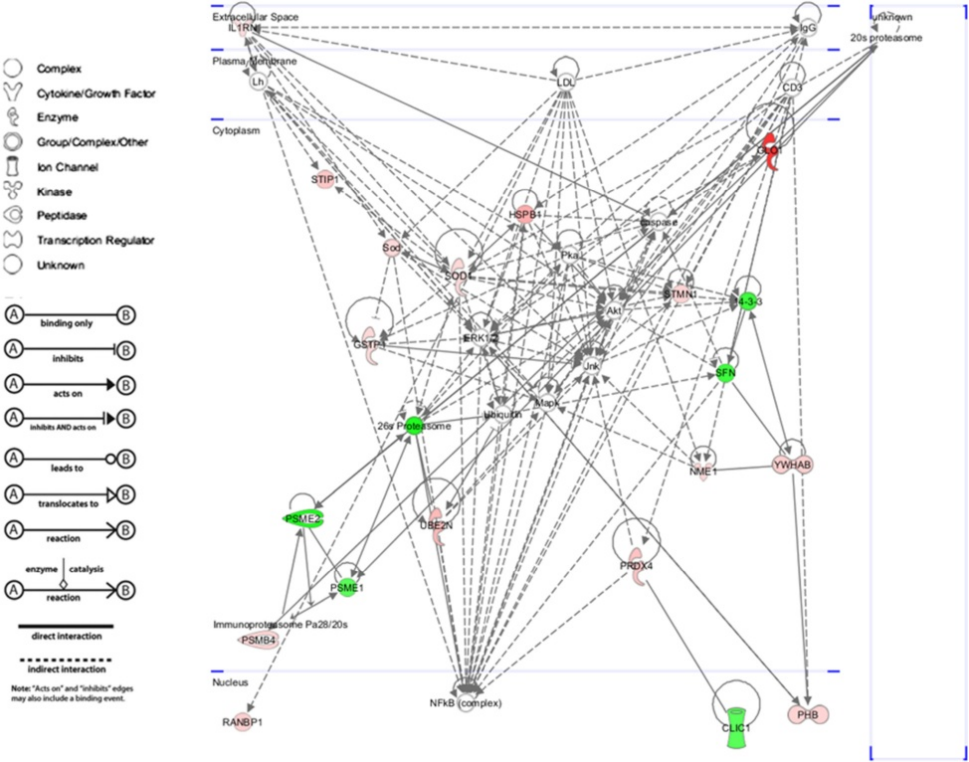
**Top-scored molecular network with identified proteins implicated in cancer progression according to the IPA software.** Proteins in the network are represented by their gene symbols. Green colored shapes denote proteins with lower abundance in oral cancer cell lines, while red colored shapes denote proteins with higher abundance in oral cancer cell lines A) molecule types, B) relationship types.

When proteins with different abundance in oral cancer were subjected to analysis using IPA, 18 were predicted to be related to cancer with involvements in hyperplasia, metastasis, invasion, growth and tumorigenesis. Table 2 shows a list of proteins known to have roles in various cancers based on IPA. Figure 2 shows a graphical representation of the predicted molecular relationships between these proteins. The predicted network involved interplay between the various proteins and suggested that changes in abundance of these proteins may influence the function of other proteins within the network. IPA also predicted that most of the proteins were of cytosolic origin.

**Table 2:** Proteins that are known to be cancer associated based on IPA

**Category**	**Functions annotation**	**p-value**	**Molecules**	**# of molecules**
Cancer	Cervical tumor	1.55E-04	GSTP1, HSPB1, PHB, TPM3	4
Cancer	Breast cancer	1.82E-04	GGCT, GLO1, GSTP1, NME1, PHB, RANBP1, SOD1, TPI1	8
Cancer	Nodular hyperplasia of liver	1.25E-03	SOD1	1
Cancer	Hematological neoplasia	1.89E-03	CCT6B, GSTP1, NME1, PSME1, STIP1, STMN1	6
Cancer	Lymphohematopoietic cancer	2.21E-03	CCT6B, GSTP1, NME1, PSME1, STIP1, STMN1	6
Cancer	Cervical cancer	2.44E-03	GSTP1, HSPB1, TPM3	3
Cancer	Multiplicity of benign tumor	2.50E-03	GSTP1	1
Cancer	Neuroblastoma	3.33E-03	GSTP1, NME1	2
Cancer	Epidermal hyperplasia	4.24E-03	PHB, RANBP1	2
Cancer	Metastasis of melanoma	4.99E-03	IL1RN	1
Cancer	Gastric cancer	5.75E-03	GSTP1, IL1RN, PHB	3
Cancer	Digestive organ tumor	6.00E-03	GSTP1, IL1RN, NME1, PHB, SFN, SOD1, STIP1, TPI1	8
Cancer	Invasion of trophoblast	7.48E-03	NME1	1
Cancer	Urothelial bladder carcinoma	7.48E-03	GSTP1	1
Cancer	Non-small cell lung cancer	7.89E-03	GSTP1, MYL12A, STMN1	3
Cancer	Cancer	8.08E-03	CCT6B, GGCT, GLO1, GSTP1, HSPB1, IL1RN, MYL12A, NME1, PHB, PSME1, RANBP1, SFN, SOD1, STIP1, STMN1, TPI1, TPM3	17
Cancer	Liver cancer	9.35E-03	GSTP1, NME1, SOD1, TPI1	4
Cancer	Immortalization of keratinocytes	9.96E-03	SFN	1
Cancer	Invasion of extracellular matrix	1.12E-02	NME1	1
Cancer	Neoplasia of cells	1.21E-02	NME1, PHB, SFN	3
Cancer	Hematologic cancer	1.27E-02	GSTP1, NME1, PSME1, STIP1	4
Cancer	Hereditary diffuse gastric cancer	1.37E-02	IL1RN	1
Cancer	Growth of secondary tumor	1.49E-02	IL1RN	1
Cancer	Incidence of hepatocellular carcinoma	1.49E-02	SOD1	1
Cancer	Quantity of papilloma	1.61E-02	GSTP1	1
Cancer	Metastasis of cells	1.71E-02	NME1, PHB	2
Cancer	Hepatocellular carcinoma	2.21E-02	NME1, SOD1, TPI1	3
Cancer	Follicular adenoma	2.23E-02	GSTP1	1
Cancer	Waldenstrom’s macroglobulinemia	2.26E-02	NME1,STIP1	2
Cancer	Acute myeloid leukemia	2.31E-02	GSTP1, PSME1	2
Cancer	Tumorigenesis of colon cancer cell lines	2.35E-02	SFN	1
Cancer	Metastasis of cancer cells	2.96E-02	PHB	1
Cancer	Infection of cervical cancer cell lines	3.05E-02	PSME2, RANBP1, STIP1	3
Cancer	Growth of melanoma	3.08E-02	IL1RN	1
Cancer	Metastasis of melanoma cell lines	3.20E-02	NME1	1

### Coordinate regulation of proteins with similar function

Two groups of proteins - detoxification enzymes and proteasome activator complex - appeared to show coordinate change in protein abundance in oral cancer.

### Detoxification enzymes

Four enzymes involved in the protection of cells against oxidative stress - Lactoylglutathione lyase (LGUL), Peroxiredoxin-4 (PRDX4), Glutathione S-transferase P (GSTP1) and Superoxide dismutase (SODC) - were found to be in higher abundance in oral cancer. Malignant cells are in general under increased oxidative stress [6-8]. Thus, an increase of enzymes involved in protection against oxidative stress may be a mechanism to tolerate such stress.

LGUL Catalyzes the conversion of hemimercaptal, formed from methylglyoxal and glutathione, to S-lactoylglutathione. Inhibitors of LGUL have been proposed as possible antitumor agents that function by inducing elevated levels of methylglyoxal in cancer cells. Methylglyoxal has been shown to inhibit the growth of human leukaemia 60 cells in culture [9]. It has also been shown that LGUL inhibitors may also be a drug resistance-reversing agent [10]. Increase of LGUL level has been found in drug-resistant tumor cells and in invasive ovarian cancer and breast cancer [11].

PRDX4 is involved in the redox regulation of the cell. The level of peroxiredoxins (in particular PRDX3, PRDX4 and PRDX5) has been shown to be increased in cancer, suggesting the induction of PRDX as a response to increased production of reactive oxygen species in carcinomatous tissue [12].

GSTP1 conjugates reduced glutathione to a wide number of exogenous and endogenous hydrophobic electrophiles. It plays an important role in the protection of cells from the products of oxidative stress as well as from several environmental carcinogens. The GSTP1 gene has been shown to be overexpressed in many human tumors [13].

SODC destroys radicals which are normally produced within the cells and which are toxic to biological systems. The level of SODC has been shown to increase in a number of cancers [14-16].

### Proteasome activator complex

Proteasome activator complex subunit 1 (PSME1) and proteasome activator complex subunit 2 (PSME2) are implicated in immunoproteasome assembly and is required for efficient antigen processing. IPA showed that both proteins also interact and influence the function of the 26 s and 20 s proteaseome. The proteasome is a multicatalytic proteinase complex that is responsible for the degradation of most intracellular proteins, including proteins that are crucial to cell cycle regulation and programmed cell death, or apoptosis. Targeting the proteasome is being investigated as a useful anticancer strategy [17]. PSME has also been proposed as a potential biomarker for ovarian cancer [18]. Interestingly, our proteomics analysis showed a decrease in abundance of these proteins, whereas the proteoasome activity is expected to increase in most cancers.

### Proteins with higher abundance in oral cancer cell line

#### ***Structural protein***

Stathmin (STMN1) is involved in the regulation of the microtubule filament system where it prevents the assembly and promotes disassembly of microtubules. It has been shown to be present at high levels in a variety of human cancers and has been proposed as a potential target in cancer therapies that disrupt the mitotic apparatus [19].

#### ***Enzymes***

A number of enzymes were found to be in higher abundance in cancer cell lines. Triosephosphate isomerase (TPIS) is a glycolysis enzyme that catalyzes the reversible interconversion of triosephosphate isomers - dihydroxyacetone phosphate and D-glyceraldehyde 3-phosphate. TPIS may be associated with cancer metastasis [20].

Nucleoside diphosphate kinase A (NDKA) plays a major role in the synthesis of nucleoside triphosphates other than ATP. NDKA is involved in cell proliferation, differentiation and development, signal transduction, G protein-coupled receptor endocytosis, and gene expression. It has been shown to act as both a metastasis suppressor and promoter in different tumors [21].

Ubiquitin-conjugating enzyme E2 N (UBE2N) mediates the transcriptional activation of target genes. The increased abundance of UBE2N and other ubiquitin conjugating enzymes has been reported in various cancers [22-25].

Gamma-glutamylcyclotransferase (GGCT) catalyzes the formation of 5-oxoproline from gamma-glutamyl dipeptides and may play a significant role in glutathione homeostasis. It induces the release of cytochrome c from the mitochondria resulting in the induction of apoptosis. GGCT may be associated in the induction of apoptosis in leukemia U937 cells [26].

Proteasome subunit beta type 4 (PSB4) belongs to the proteasome multicatalytic proteinase complex. This complex is characterized by its ability to cleave peptides with Arg, Phe, Tyr, Leu, and Glu adjacent to the leaving group at neutral or slightly basic pH.

### Regulatory proteins

14-3-3 protein beta/alpha (1433B) is an adapter protein implicated in the regulation of a large spectrum of both general and specialized signaling pathways. 1433B binds to a large number of partners, usually by recognition of a phosphoserine or phosphothreonine motif. Binding will generally result in the modulation of the activity of the binding partner.

Prohibitin (PHB) has a role in regulating cell proliferation where it inhibits DNA synthesis. It has been shown to be involved in a number of different cancers [27-30].

Stress-induced-phosphoprotein 1 (STIP1) mediates the association of the molecular chaperones HSC70 and HSP90. STIP1 has been shown to be secreted by, and induces proliferation in glioma cells [31]. It has also been implicated in a number of other cancers [32-34].

Interleukin-1 receptor antagonist protein (IL1RA) inhibits the activity of IL-1 by binding to its receptor. The level of IL1RA has been shown to change in cancer [35-37].

Rho GDP-dissociation inhibitor 1 (GDIR) regulates the GDP/GTP exchange reaction of the Rho proteins by inhibiting the dissociation of GDP from them, and the subsequent binding of GTP to them. In cancer, GDIR has been implicated in metastasis, mediation of cancer cell motility and drug resistance [38-40].

Ran-specific GTPase-activating protein (RANG) inhibits GTP exchange on Ran and plays an essential role in nuclear transport by permitting RanGAP-mediated hydrolysis of GTP on Ran complexed to karyopherin b [41].

#### ***Chaperones***

Heat-shock protein beta-1 (HSPB1) is involved in stress resistance and actin organization. Heat shock proteins (HSPs) are a large and heterogeneous group of chaperones whose synthesis can be induced by both physiological and pathological conditions, such as heat shock, oxidative stress, mitogenic signals, inflammation, infection and neoplastic transformation. Although a number of HSPs has been implicated with cancer, association of HSPB1has not been reported.

### Proteins with lower abundance in oral cancer cell line

#### ***Structural proteins***

Tropomyosin alpha-3 chain (TPM3) binds to actin filaments in muscle and non-muscle cells. In association with the troponin omplex, it plays a central role in the calcium dependent regulation of vertebrate striated muscle contraction.

Myosin regulatory light chain-2 (ML12A) is a myosin regulatory subunit that plays an important role in regulation of both smooth muscle and non-muscle cell contractile activity via its phosphorylation. ML12A has been implicated in cytokinesis, receptor capping, and cell locomotion. Phosphorylation of ML12A has been shown to be critical in the invasiveness of metastatic cancer cells [42].

#### ***Regulatory protein***

14-3-3 protein sigma, also known as stratifin (1433S) is an adapter protein implicated in the regulation of a wide variety of both general and specialized signaling pathways. 1433S is a potential lymph node metastasis-related protein in lung squamous carcinoma [43].

#### ***Chaperones***

T-complex protein 1 subunit zeta-2 (TCPW) is a molecular chaperon that assists in the folding of proteins upon ATP hydrolysis [44].

#### ***Others***

Chloride intracellular channel protein 1 (CLIC1) is a protein that can insert into cellular membranes to form chloride ion channels. It has been shown that overexpression of CLIC1 promoted cell motility and invasion of low metastasic cell lines (GBC-SD18L) *in vitro*, while RNA interference of CLIC1 decreased cell motility and invasive potency of highly metastasic cell lines (GBC-SD18H) *in vitro*, suggesting that CLIC1 may play an important role in metastasis of gallbladder carcinoma [45]. CLIC1 has also been proposed as a potential biomarker [46,47] and therapeutic target [48] for cancer.

### Assessment of mRNA transcript

**Figure 3:**
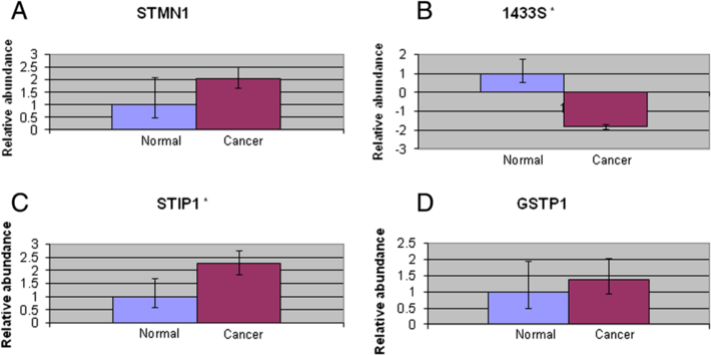
**Results of quantitative RT-PCR showing relative expressions of STMN1, 1433S, STIP1 and GSTP1.** Total mRNA was extracted from normal and oral cancer cell lines. Quantitative RT-PCR was then performed as described. **A**: Relative expression of STMN1, **B**: Relative expression of 1433S, **C**: Relative expression of STIP1, **D**: Relative expression of GSTP1. Relative intensities of all genes of interest were determined by using β-actin as an internal standard. Results represent the mean ± SD for three experiments. *p < 0.05.

Quantitative real-time PCR was performed to assess the mRNA expression of four proteins - STMN1, 1433S, STIP1 and GSTP1. These proteins were selected due the marked difference in their abundance and/or possible involvement in cancer. They also represented proteins from various functional categories. The mRNA expression of all four genes was found to correlate with its corresponding protein abundance. However, only 1433S and STIP1 was found to be statistically significant (Figure 3). This suggested that these proteins may be regulated at the mRNA level.

## Conclusions

We have showed that a comparative study of the oral cancer cell line proteome may identify cancer associated proteins. IPA predicted that at least 18 of these proteins were associated with cancer development and progression. At least two identified proteins (CLIC1 and PSME) have previously been proposed as potential biomarkers for cancer. Two groups of proteins (detoxification enzymes and proteasome activator complex) appeared to show coordinate change in protein abundance. Taken together, these results has demonstrated the potential of this type of study as an alternative to directly analyzing tissue samples in identifying cancer associated proteins. This study can be further extended by investigating the presence/absence of these proteins in tissue samples.

## Methods

### Establishment of cell cultures

This project was approved by the Medical Ethics Committee, Faculty of Dentistry, University of Malaya and endorsed by the Ministry of Health, Malaysia (DFOP1006/0041[P]). Normal tissues were derived from surgically resected tissue specimens from different impacted tooth patients. Cancerous tissues were derived from surgically resected tissue specimens from three different oral squamous cell carcinoma patients. Sample collection was performed at the Faculty of Dentistry and University Malaya Medical Centre, Kuala Lumpur Hospital and Tengku Ampuan Rahimah Hospital. All tissues were acquired after written informed consent was obtained from the patients. The oral cancer cell lines and primary cultures were derived from squamous cells and were originated from the same anatomic site (gum). Cell cultures were established as previously described [49,50].

### Sample preparation and protein extraction

Cells were grown to confluence. Following trypsinization, cells were pelleted and washed 3× with PBS. The cells were then lysed by repeated freeze thawing in extraction buffer (5 M Urea, 2 M Thiourea, 2% SB3-10 and 2% CHAPS). Protein concentration was determined by Bradford assay using Bovine Serum Albumin as standard according to the manufacturer’s instruction (Bio-Rad Laboratories).

### Two-Dimensional Gel Electrophoresis (2DE)

For each cell line/primary culture, 2DE was performed in triplicate on protein samples extracted from three different passages of growth. Isoelectric focusing for 2DE was performed using an IPGphor system (Amersham Biosciences) according to the manufacturer’s protocol. Briefly, IPG strips (24 cm Immobiline IPG Drystrip pH 4–7) were rehydrated overnight using 450 μl/strip urea rehydration stock solution (8 M Urea, 2% w/v CHAPS, 0.5% v/v Pharmalyte/IPG Buffer pH3-10, 1% trace bromophenol blue) without containing the sample. Protein extracts were solubilized in 80 μl sample lysis buffer (8 M Urea, 4% w/v CHAPS, 40 mM Tris, 65 mM DTT, 1% trace bromophenol blue) and loaded into the loading cup just before running. A total of 80 μg protein was loaded for analytical gels and 160 μg loaded for preparative gels. Isoelectric focusing was performed for 60 kVh with running conditions as follows: 500 V: 1 hour, 1000 V: 1 hour, 1000 V: 3 hours and finally a constant 8000 V at 20°C.

For the second dimension, the IPG strips were first equilibrated in 10 ml of SDS equilibration buffer (6 M Urea, 75 mM Tris–HCl pH8.8, 87% w/w glycerol, 2% SDS, 1% trace bromophenol blue) containing 1% DTT for 10 minutes, followed by a second equilibration for 10 minutes in the same equlibration buffer containing 2.5% Iodoacetamide. Equilibrated strips were then placed on 11% SDS-Polyacrylamide gel and enclosed with 0.5% agarose gel. Second dimension electrophoresis of reduced and alkylated samples was carried out using an Ettan Dalt twelve system (GE Healthcare). Electrophoresis was initially performed at 2 W for 45 minutes, followed by 110 W until the bromophenol blue dye reached the bottom edge of the gel.

Protein spots were visualized using protocols described in the PlusOne™ Silver staining kit (GE Healthcare). The complete protocol was followed for analytical gels. For preparative gels, a modified protocol was used. Glutaraldehyde was omitted from the sensitization step and formaldehyde omitted from the silver reaction step [51].

### Gel image analysis

Following 2DE and silver staining, gels were scanned (Image Scanner III, GE Healthcare) and protein profiles compared using the ImageMaster Platinum 7.0 software (GE Healthcare). Protein spots were normalized using percentage volume. Statistical analysis for the comparison of protein abundance between the groups was performed by Student’s t-test. Only protein spots with fold difference > 1.4 and p < 0.05 were considered to have significantly changed in abundance.

### Tryptic digestion

Protein spots were excised and in-gel digested with trypsin (Promega) for mass spectrometric identification according to published protocols [52-54].

### MALDI-TOF/TOF mass spectrometry analysis and database searching

Protein identification was performed as previously described using a MALDI-TOF mass spectrometer (ABI 4800 Plus, Applied Biosystems) [55,56].

### Bioinformatics

Categorization of protein function was determined based on Swiss-Prot/TrEMBL database search. Ingenuity Pathway Analysis (IPA, http://www.ingenuity.com) was used to determine the localization of identified proteins and their participation in molecular networks involved in carcinogenesis according to the well-established Ingenuity Knowledge Base. Details regarding proteins that were different in abundance were exported to the IPA software. Each protein identifier was then mapped to its corresponding protein object and was overlaid onto a global molecular network developed from information contained in the Ingenuity Knowledge Base. Protein networks were then algorithmically generated based on their connectivity. A Right-tailed Fischer’s exact test was used to calculate a p-value indicating the probability that each biological function assigned to the network is not due to chance alone.

### Real-time PCR (RTPCR)

All experiments were performed according to manufacturers’ instructions. Total mRNA was extracted from cell pellets using RNAqueous®-4PCR Kit (Ambion). High Capacity cDNA Reverse Transcription kit (Applied Biosystems) was used to reverse transcribe total mRNA into cDNA templates. RTPCR was performed by either Taqman® Gene Expression Assay or Fast Sybr Green (Applied Biosystems).

Primers were either designed using the Primer3 software or were based on literature and PrimerBank [57,58]. The specificity of primers was examined using NCBI primer-Blast (http://www.ncbi.nlm.nih.gov/tools/primer-blast/). All PCR reactions were performed using the StepOnePlus Real-Time PCR instrument (Applied Biosystems). PCR conditions were defined according to the manufacturer’s recommended parameters. Gene expression levels were comparatively analyzed using StepOne software v2.2. Raw data from the experiments were recalculated as a mean expression level of cancer and normal groups to generalize the results of comparisons between the two groups. Student’s unpaired t-test was then performed to determine the significance of the results.

## Competing interest

The authors declare that they have no competing interests.

## Authors’ contributions

SAK - principal investigator, designed and conceived the experiments, performed all MALDI-TOF/TOF analysis and prepared the manuscript. NAS - performed the experiments. RBZ - provided tissue samples from which cell lines were derived. SCC - established the cell cultures. MAR – designed and conceived the experiments and prepared the manuscript. All authors read and approved the final manuscript.
